# Outpatient Tubeless Percutaneous Nephrolithotomy Performed in a Freestanding Ambulatory Surgery Center

**DOI:** 10.1089/cren.2017.0136

**Published:** 2018-02-01

**Authors:** Joel E. Abbott, Julio G. Davalos

**Affiliations:** Department of Surgery, University of Maryland School of Medicine, Baltimore, Maryland.; Advanced Kidney Stone Center of the Americas, Chesapeake Urology Associates, Hanover, Maryland.

**Keywords:** percutaneous nephrolithotomy, percutaneous renal surgery, renal stone, urolithiasis

## Abstract

***Background:*** Percutaneous nephrolithotomy (PNL) is a procedure that has traditionally been performed in an inpatient or hospital setting. Many surgical procedures have evolved over time from an inpatient/hospital setting to outpatient procedures performed in surgical centers. Outpatient PNL has become an accepted standard in select patients, but to date, the procedure has not been performed in an outpatient surgical center.

***Case Presentation:*** We describe our initial experience managing large renal stone burden with PNL performed completely outpatient in a freestanding ambulatory surgery center. The patient was carefully selected as a young, healthy, thin patient with straightforward renal stone burden and favorable anatomy per CT. Access was achieved with a combination of fluoroscopic and endoscopic needle guidance. The procedure was performed with several modifying factors to enable an effective outpatient discharge.

***Conclusion:*** Our experience reinforces the outpatient feasibility of PNL and incites the possibility of transitioning the procedure to an ambulatory surgical center in select patients to provide healthcare savings and an improved patient experience.

## Introduction

Percutaneous nephrolithotomy (PNL) was first performed in 1976. Ten years later, Preminger et al. demonstrated the feasibility of performing the procedure outpatient in select five patients discharged from the hospital same day with nephrostomy tubes in place.^1^ This idea resurfaced when Darren Beiko forefronted the contemporary shift toward tubeless PNL performed outpatient and subsequently published the largest cohort to date (*n* = 50) in 2015.^2,3^ To our knowledge, outpatient tubeless PNL in a freestanding ambulatory surgery center (ASC) has not been reported in the literature to date. We describe our initial experience with ASC-based PNL.

Our objective with this pilot initiative is to assess the feasibility of performing PNL in a freestanding ASC, challenging the traditional standard of performing percutaneous renal surgery in a hospital with postoperative hospitalization, and to report our experience with PNL in the ASC. We hypothesize that in properly selected patients, PNL is a procedure that can be safely and effectively performed in a freestanding ASC without compromising patient safety.

## Case Presentation

**FIG. 1.**
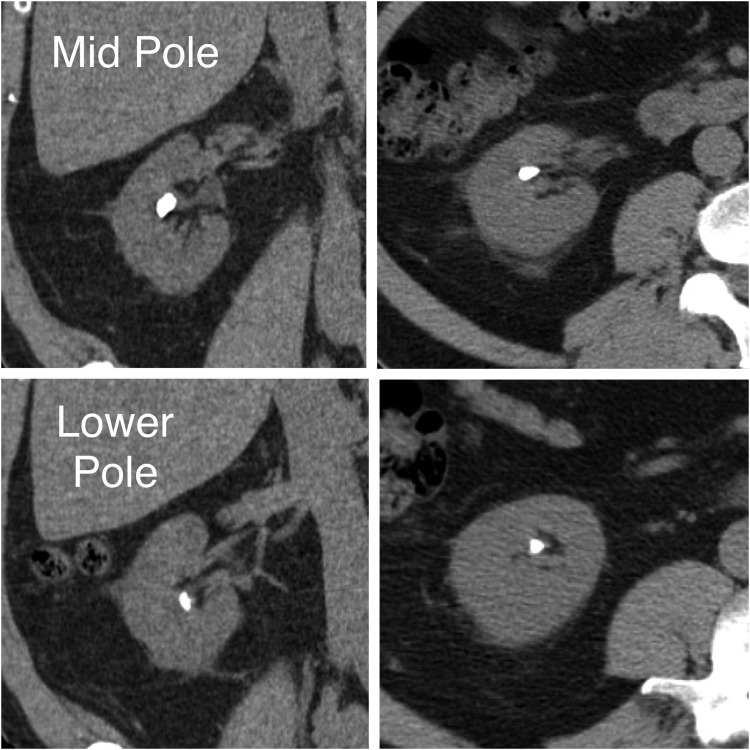
CT demonstrating patient's intra-abdominal anatomy and stone burden.

This is a 59-year-old male referred to the Advanced Kidney Stone Center of the Americas, Division of Chesapeake Urology, on February 24, 2015, with bilateral nonobstructing large renal calculi, initially diagnosed during hematuria work-up, who was interested in stone removal by PNL. CT showed the right kidney with a 10 × 9 mm stone in the mid-pole calix and a 6 × 5 mm stone in the lower pole calix, and the left kidney with four stones in the lower pole calices, the largest of which measures 9 × 8 mm (Fig. 1). All management options were discussed and provided to the patient, including observation, shock wave lithotripsy, ureteroscopic approach, and percutaneous techniques. The patient opted for bilateral percutaneous management, wanting to minimize stone fragmentation and maximize stone clearance; therefore, a staged percutaneous approach was planned for treatment of his bilateral stones. Review of medical and surgical history demonstrated that he was a candidate for outpatient PNL. On April 13, 2015, he underwent left PNL performed outpatient (same-day discharge from the postanesthesia care unit) at University of Maryland Baltimore Washington Medical Center. He was subsequently scheduled for right side outpatient PNL to be performed on April 20, 2015, in a freestanding ASC (not affiliated or attached to a hospital). We describe this procedure performed in our ASC.

### Procedure

**FIG. 2.**
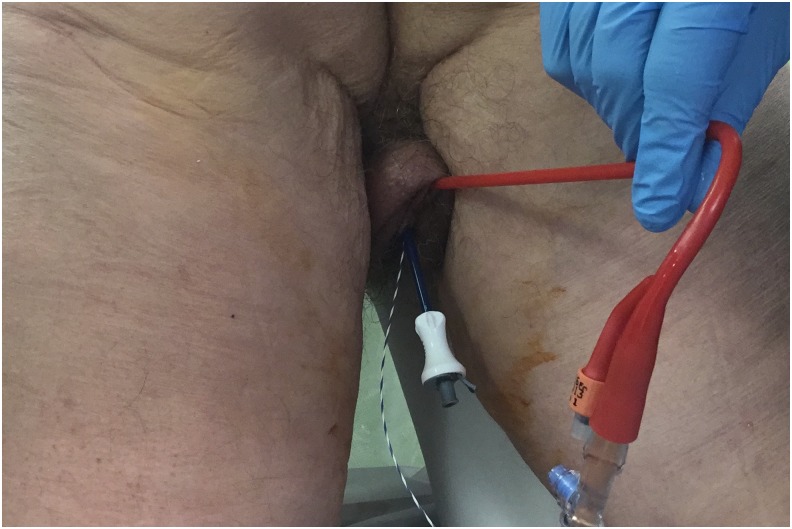
Transurethral placement of bladder catheter, ureteral access sheath, and safety wire in preparation for ambulatory percutaneous nephrolithotomy.

**FIG. 3.**
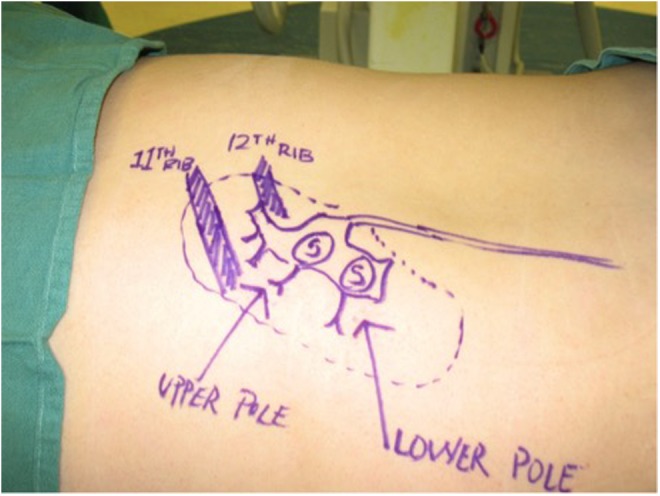
Example of our “skin template” created under fluoroscopic guidance before access showing the patient's renal anatomy and stone burden. This is routinely performed for percutaneous renal cases (Note: photo shown is a left side procedure).

**FIG. 4.**
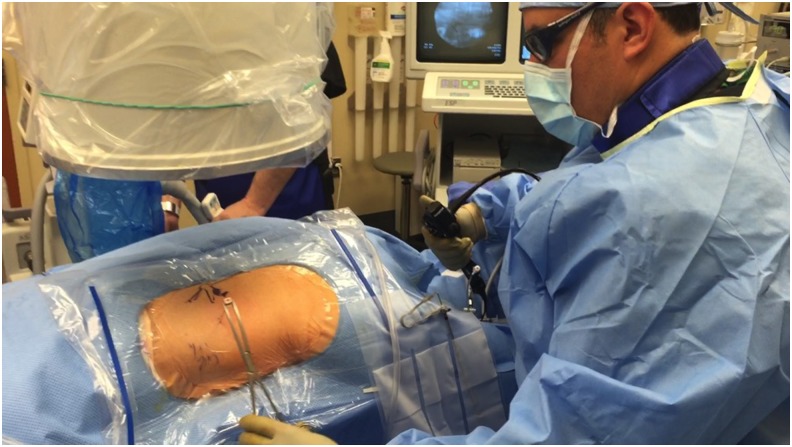
Example of surgeon performing renal access utilizing a combination of ureteroscopic guidance and fluoroscopic radiographic guidance. (Note: photo shown is a left side procedure).

The patient was taken to the operative suite and anesthetized on the gurney in supine position. Flexible cystoscopy was then performed and the operative side ureteral orifice was cannulated with a 0.035 inch Sensor Wire (Boston Scientific, Boston, MA), advanced up the ureter to the renal pelvis. The cystoscope was withdrawn and removed from the patient leaving the wire in place. The cystoscope was then brought back into the bladder adjacent to the wire. The ureter was then sequentially dilated over the wire utilizing a 5F open-end ureteral catheter (Boston Scientific) followed by an 8F to 10F coaxial dilator. Leaving the 10F sheath in place, a second wire was advanced up the ureter to serve as a safety wire. A Zebra Wire (Boston Scientific) served as the safety wire, preferred because it is a different color scheme and more radiolucent than the sensor wire preventing confusion with antegrade working wires. Continued sequential ureteral dilation was performed with the 11F inner portion of the access sheath. An 11/13 Navigator (Boston Scientific) ureteral access sheath (UAS) was then easily placed under cystoscopic visualization and without the need for fluoroscopy. CT scan was previously reviewed to ensure no ureteral stones. Cystoscopic-guided UAS placement would be aborted if resistance is felt and alternatively, fluoroscopy guidance or ureteroscopy would be employed. A urethral catheter was then placed into the bladder over wire adjacent to the UAS to provide both ureteral access and bladder drainage (Fig. 2).^4^ Patient was then transferred from the gurney to the operating table positioned prone on an Allen Advance “Jackson Spinal” Table (Allen Medical, Acton, MA), surgical table equipped with a Allen Bow Frame (Allen Medical). Scout fluoroscopy images are obtained followed by retrograde pyelography. A marking pen was used to create a skin template of the renal anatomy and surrounding structures drawing the ribs, kidney stones, renal collecting system, and pleura (location previously determined with careful review of the CT scan) (Fig. 3). Retrograde ureterorenoscopy was performed to evaluate the stones and identify preferred caliceal access for stone treatment. Air bubbles and C-arm rotations help to identify posterior calices. A 12.5 cm 18-gauge needle (Boston Scientific) was used in obtaining access (Fig. 4) and subsequently cannulated with a 0.035 in Sensor Wire positioned into the renal collecting system. Skin incision was made with 11-blade scalpel. Sequential dilation of the tract was then performed with an 8F to 10F coaxial dilator (Boston Scientific). A second Sensor Wire is placed to serve as a safety. A 30F 12 cm NephroMax balloon with 30F/34F 17 cm clear renal sheath (Boston Scientific) was used to dilate the tract and maintain renal access during the procedure. All percutaneous maneuvers in achieving access, including needle and wire entry and tract dilation, were visualized endoscopically to provide an additional layer of safety and precision during these critical steps. Renoscopy and ureteroscopy were performed with a standard rigid nephroscope and flexible ureteroscope (Karl Storz, Tuttingen, Germany). Lithoclast (Boston Scientific) device and Perc-NCircle (Cook Medical, Bloomington, IN) stone retrieval device were employed in extracting stones. In addition to the stones visualized on CT, another two stones (2 and 3 mm) were identified endoscopically and extracted. Final endoscopic evaluation of the kidney was performed with a combination of antegrade and retrograde flexible scope renoscopy. Final ureteroscopy was performed as the ureteroscope and UAS were withdrawn in tandem. At this point, the urethral catheter was clamped to permit slight bladder dilation and the ureteroscope was positioned within the bladder, visualizing the operative side ureteral orifice. Ureteral stent was then deployed over wire appropriately positioned, coiled within the bladder, and renal pelvis visualized both endoscopically and radiographically. A dissolvable hemostatic plug was advanced down the renal access sheath into the renal access tract before removal of the access sheath.^5^ Multilevel (11th and 12th) intercostal anesthetic nerve blocks are performed using 0.5% Marcaine. Incision was closed with 2–0 Vicryl followed by 3–0 Monocryl and skin glue. Anesthetic was reversed and patient was transported to the postoperative recovery area. Acetaminophen 1000 mg and ketorolac 30 mg were both given intravenous before leaving the operative suite. Total time in odds ratio was 1 hours and 57 minutes. Total fluoroscopy time was 42 seconds. Renal access time was one minute. Fifty milliliters blood loss was recorded. Patient was subsequently discharged home in <90 minutes.

## Discussion

The prevalence of kidney stone disease and incidence of stone-related events have demonstrated a constant rise in recent era. Utilization of ambulatory surgical centers as the site of service for treatment of these stones has demonstrated equivalent quality of care and a significant cost-saving method to the healthcare system compared to procedures performed in the hospital.^6^ In addition, ASCs can provide improved surgeon efficiency without detracting from quality of care or patient satisfaction.^7,8^ Ambulatory PNL (aPNL) (most often performed tubeless) has been shown to be a safe procedure in select patient with a high stone clearance rate and low complication and readmission rate.^1^ Following the trend of other surgical procedures deemed safe and appropriate to be performed outpatient, it only seems appropriate that we consider select patients for an aPNL in the ASC. We have demonstrated this potential in performing the first reported aPNL in a freestanding surgical center without access to hospital resources.

Several procedural refinements have been employed to facilitate aPNL. Most important is a mastery of renal access by the operative urologist, avoiding the common practice of interventional radiologist-performed renal access that often proves to be suboptimal for stone treatment. This practice not only adds to patient discomfort but also can limit the ability of the urologist to clear stone when utilizing an access point into the kidney that is not ideally located.

Ureteroscopy is performed before access to endoscopically evaluate stone location and collecting system anatomy. It facilitates precise access into preferred calix for stone treatment. Access is obtained utilizing both fluoroscopic and endoscopic simultaneous guidance. This is thought to improve safety and precision. Precise renal access improves stone-free rates and minimizes renal trauma.

After stone clearance, a ureteral stent is placed and a hemostatic plug is deployed into the tract before wound closure to facilitate tamponade of the surgical tract.^2^ Every effort is made to not place a nephrostomy tube unless absolutely clinically indicated—such as the presence of pyonephrosis or significant collecting system disruption. Multilevel (11th and 12th) intercostal anesthetic nerve blocks are performed using 0.5% Marcaine. Acetaminophen and ketorolac are administered intravenously before concluding the procedure. These efforts lead to the patients' postoperative pain profiles closely mimicking that of postureteroscopy, with stent colic and catheter discomfort as primary complaints. Foley catheter is removed in the recovery room to reduce catheter irritation. Patient satisfaction and experience compared to the “traditional” approach were far superior.

## Abbreviations Used

aPNL: ambulatory PNL
ASC: ambulatory surgery center
PNL: percutaneous nephrolithotomy
UAS: ureteral access sheath
